# Zebrafish as a Model for Cardiovascular and Metabolic Disease: The Future of Precision Medicine

**DOI:** 10.3390/biomedicines12030693

**Published:** 2024-03-20

**Authors:** Ramcharan Singh Angom, Naga Malleswara Rao Nakka

**Affiliations:** Department of Biochemistry and Molecular Biology, Mayo Clinic College of Medicine and Science, 4500 San Pablo Road South, Jacksonville, FL 32224, USA; nakka.naga@mayo.edu

**Keywords:** cardiovascular disease, metabolic disease, transgenesis, zebrafish, gene editing, CRISPR, TALEN

## Abstract

The zebrafish (*Danio rerio*) has emerged as an appreciated and versatile model organism for studying cardiovascular and metabolic diseases, offering unique advantages for both basic research and drug discovery. The genetic conservation between zebrafish and humans and their high fecundity and transparent embryos allow for efficient large-scale genetic and drug-oriented screening studies. Zebrafish possess a simplified cardiovascular system that shares similarities with mammals, making them particularly suitable for modeling various aspects of heart development, function, and disease. The transparency of zebrafish embryos enables the real-time visualization of cardiovascular dynamics, offering insights into early embryonic events and facilitating the study of heart-related anomalies. In metabolic research, zebrafish provide a cost-effective platform for modeling obesity, type 2 diabetes, hyperlipidemia, and other metabolic disorders. Their high reproductive rate allows for the generation of large cohorts for robust statistical analyses, while advanced genetic tools, such as CRISPR/Cas9, enable precise gene editing with which to model specific genetic mutations associated with human diseases. Zebrafish metabolic models have been instrumental in elucidating the molecular mechanisms underlying metabolic diseases, studying the effects of environmental factors, and identifying potential therapeutic targets. Additionally, the permeability of zebrafish embryos to small molecules facilitates drug discovery and screening, offering a rapid and economical approach to identifying compounds with therapeutic potential. In conclusion, zebrafish cardiovascular and metabolic disease models continue to contribute significantly to our perception of disease pathogenesis, providing a platform for translational research and developing novel therapeutic interventions. The versatility, scalability, and genetic manipulability of zebrafish position them as an invaluable asset in unraveling the complexities of cardiovascular and metabolic diseases. This review presents an overview of the zebrafish model’s key features and contributions to investigating cardiovascular and metabolic disorders. We discuss the benefits and drawbacks of using zebrafish models to study human disease and the critical findings revealed by the progress in this endeavor to date.

## 1. Introduction

The Zebrafish was introduced as an animal model for use in genetic investigations, by Streisinger and colleagues [1]. Large-scale phenotypic screening using N-ethyl-N-nitrosourea (ENU) mutagenesis has identified several mutants [2,3,4]. Nevertheless, following forward genetic screening, the positional cloning of every ENU mutation required much time and effort [5]. Advanced gene-targeting techniques using zinc-finger nucleases (ZFNs), transcription activator-like effector nucleases (TALENs), and clustered regularly interspaced short palindromic repeats/CRISPR associated protein 9 (CRISPR/Cas9) have surmounted difficulties in producing targeted gene knockout alterations. CRISPR/Cas9 provides knockout animals for zebrafish researchers through a practical reverse genetic approach [6,7,8,9,10,11,12].

**Figure 1:**
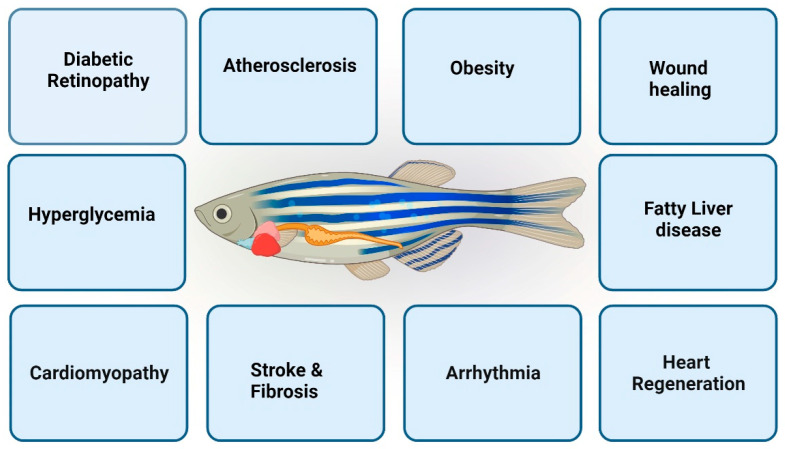
Outline showing various applications of zebrafish in the field of cardiovascular disease and metabolic disease.

Furthermore, the use of zebrafish for understanding human genetic illnesses has been made easier by the high degree of genome structural similarity shared between humans and zebrafish (~70% of human genes have at least one evident zebrafish ortholog, while this value is 80% of human genes with respect to mouse orthologs) [13,14,15]. Zebrafish are increasingly being used to uncover the underlying links between the genotypes and phenotypes of numerous human diseases (Figure 1) thanks to recent developments in next-generation sequencing (NGS) and the need for tailored therapy. Zebrafish also have several advantages, including their amenability to imaging of their developing embryos at the organ level due to optical clarity and the fact that they contain hundreds of embryos in a single cluster [16,17]. Additionally, it is easier to create tissue-specific transgenic animals using a variety of well-chosen gene promoters. Through linking with regulatory components like GAL4/UAS or Cre/LoxP, the Tol2-based transgenic system in zebrafish has recently improved, allowing for the spatiotemporal regulation of gene expression [17,18,19]. These advantages make it possible to watch cellular dynamics in vivo and image cells in real-time, which helps us to study the underlying molecular mechanisms of different developing organs and explore the disease in vertebrates.

## 2. Genetic Studies Using Zebrafish

Forward and reverse genetic techniques have been effectively applied to zebrafish to discover new signaling pathways and investigate gene functions. Two extensive forward genetic screens have found hundreds of mutant phenotypes related to different facets of development and embryogenesis [4,20]. Without prior knowledge of the underlying genes, such mutants have offered fresh perspectives on a wide range of intricate biological processes. Characterizing these mutants and discovering the mutated genes that produced them helped clarify the cellular and molecular causes of several human diseases since many zebrafish mutant phenotypes are like human hereditary diseases. In another landmark development, more than 50 mutant lines related to cardiac development or function were identified in early screens [21,22]. Multiple genetic regulators of the signaling cascade that are crucial for cardiovascular development were identified in these screens. Since then, several transgenic-assisted screens have been conducted [23], with a focus on certain phases of cardiac morphogenesis.

## 3. Methods

In order to obtain unbiased and comprehensive information, Scopus, PubMed, and Google Scholar were used as search engines to retrieve published data. The keywords used for this search include “zebrafish model of cardiovascular disease, a zebrafish model of metabolic disease”. A total of 250 articles were obtained (published between 2000 and December 2023), out of which only 212 were selected based on the inclusion and exclusion criteria below.

- Inclusion criteria:
  A.1. Research articles that report on genetic or non-genetic models of cardiovascular and metabolic disease.
  A.2. Research articles that involve the keywords heart failure, cardiomyopathy, hypertrophy, arrhythmia, regeneration, drug screen, obesity, diabetes, and atherosclerosis.
  A.3. Research articles that report the genetic tools used in zebrafish to investigate cardiovascular and metabolic diseases.
- The exclusion criteria were set to exclude the following articles:
  B.1. Articles that report on other animal models apart from zebrafish.
  B.2. Articles that report on zebrafish models of other vascular diseases or other diseases.
  B.3. Articles that report on aging-related disorders.
  B.4. Article published in languages other than English.

## 4. Zebrafish as Cardiovascular Disease Models

The zebrafish (*Danio rerio*) has surfaced as a valuable model organism for cardiovascular disease (CVD) research [24,25,26], offering unique advantages for exploring the intricacies of cardiovascular development, function, and pathology (Figure 1). This section of our review delves into the strengths and limitations of the zebrafish cardiovascular disease model, shedding light on its contributions to advancing our understanding of cardiovascular disorders. In the last decade, the zebrafish has been increasingly used for various human-disease-related studies [27,28]. For decades, mice have been predominantly used as excellent models for investigating human cardiac diseases. Although mouse models have provided valuable insight into diseased cardiac states, they also have limitations in their application, as their electrophysiological characteristics and basal heart rate are highly different compared to those of humans. Also, in vivo, the investigation of mouse models often requires invasive imaging and monitoring techniques, which requires sacrificing the animal.

Furthermore, generating and maintaining mouse lines are time consuming and expensive. As an alternative model, zebrafish can complement these bottlenecks and allow for the easy study of heart development, heart rate analysis (electrophysiology), and phenotypic characterization [21,29,30,31]. Zebrafish provide an alternative solution to the study of early mutations affecting the formation and function of the heart (Table 1). This section also presents an overview of some major cardiac disorders modeled using zebrafish (Table 2). These models have been developed to potentially unearth the major genes and molecular pathways that are involved in CVD pathophysiology. Numerous aspects of heart development, function, and illness have been modeled using zebrafish.

Along with the genetic and experimental strategies used to produce and examine these models, zebrafish have contributed significantly to our understanding of heart abnormalities. Zebrafish are also helpful in identifying potential genes for congenital cardiac abnormalities. Table 2 lists some established zebrafish-based cardiovascular disease models [32,36,37,38,39,40,41,42,43,44,45,46,47].

### 4.1. Heart Regeneration

Zebrafish have shown great promise in the study of heart failure. Different species experience cardiac regeneration, which is an intriguing biological process, at different rates. Although cardiac tissue can regenerate in newborn mice, this ability disappears seven days after birth, and fibrotic scar formation replaces the damaged myocardium [48]. Zebrafish, among other vertebrates, have an innate capacity for cardiac regeneration after severe myocardial damage and, therefore, are now a great model for investigating the molecular pathways involved in the regeneration process [46,49]. Zebrafish also have a remarkable scope for cardiac muscle regeneration after sustaining a considerable injury and are widely used as models for regenerative studies, showing real promise in heart failure research, as compared to higher vertebrates, including mammals, adult zebrafish exhibit extraordinary regenerative potential and can regenerate many organs and tissues, including the retina [50], brain tissues [51], spinal cord [52], and heart [46,53,54,55]. Applying zebrafish to study cardiac tissue regeneration seems to be a practical step toward regenerative medicine. In the zebrafish heart, when 20% of the myocardium is removed, new cells differentiate from cardiac progenitor cells in a process determined by the epicardium, which appears to recapitulate many elements of embryonic cardiac development [43].

Similarly, zebrafish have also been shown to exhibit improved recovery after experimental myocardial infarction when combined with p38 MAP kinase inhibition [55]. Primary cardiomyocytes from adult zebrafish hearts can be easily isolated and cultured for up to 4 weeks and used as an alternative to in vivo experiments [56]. Several studies have focused on hypoxia’s effect on zebrafish’s regenerative capacity [57,58]. These studies on zebrafish have demonstrated the functions/effects of nerve growth factors in regard to stimulating cardiac regenerative processes in a failing heart by mediating cardiomyocyte proliferation [59].

Cryoinjuries (CIs) have been induced in zebrafish to model and study the mechanisms of heart regeneration and scar removal post-myocardial infarction [60]. A CI is an invasive procedure that more closely models the pathophysiological processes of human myocardial infarction [61]. A zebrafish model mimicking hypoxia-induced ischemic injury in the mammalian heart was developed to assess regenerative capacity [58]. Zebrafish exposed to hypoxia/reoxygenation (H/R) exhibited cardiomyocyte apoptosis and necrosis. However, cardiomyocyte proliferation was apparent 18–24 h after H/R; 2D-echocardiography was used to measure cardiac function, and the compromised ventricular function recovered to the baseline level. Thus, it is feasible to study reperfusion injury in zebrafish.

Further, in zebrafish, cardiac regeneration following myocardial infarction (MI) is a collective outcome involving multiple cell types. Various processes, including the infiltration of inflammatory cells, the development of fibrosis, the regeneration of cardiomyocytes, the regression of scar tissue, and the restoration of cardiac function, are orchestrated by endocardial, epicardial, fibroblast, and cardiomyocyte cells, among others [62]. In zebrafish, research has revealed that the epicardium supports myocardial regeneration in a cryoinjured heart. Cells derived from the epicardium (namely, EPDCs) migrate to the damaged myocardium after injury and differentiate into myofibroblasts and perivascular fibroblasts, which promote new angiogenesis through paracrine signaling [63]. In addition to the epicardium, the endocardium also plays a role in cardiac regeneration by providing soluble signals to cardiomyocytes and activating genes necessary for cardiac structure development and maintenance, such as hand2 and stat5b [64,65]. A recent study reported a single-cell transcriptomic analysis of non-CMs during cardiac regeneration in zebrafish. It provides an outline for questioning this process’s molecular and cellular basis [66].

### 4.2. Zebrafish Cardiomyopathy Models

Characterizing the key genetic basis and molecular mechanisms underlying human dilated cardiomyopathies (DCM) faces major bottlenecks, and zebrafish models of DCM provide a beneficial resource in the systematic dissection of genetic pathways and the development of better therapeutic strategies [26,67,68,69,70,71]. Titin molecules play a role in sarcomeric assembly in the heart and are crucial for maintaining myofibrillar elasticity and integrity. A mutation in the titin gene is associated with DCM in both humans and zebrafish [72,73]. The Titin gene encodes giant-muscle filament titin (TTN) and causes autosomal-dominant DCM linked to chromosome 2q31. Zebrafish has two titin orthologs, *ttna* and *ttnb,* of which *ttna* is required for group Z-discs and A-bands in the sarcomere and for cardiac contractility. Different titin isoforms have distinct functions, and their disruption leads to variable phenotypes in DCM [72]. Zebrafish runzel mutants have been linked to one of the titin loci and are homozygous lethal with a muscular dystrophy phenotype [73]. 

Gene mutations in zebrafish laminin alpha4 and integrin-linked kinase were reported in a forward mutagenesis screen. Similar mutations were over-represented in a human cohort with DCM [74,75]. A knockdown of the zebrafish transcriptional co-activator eyes absent homolog 4 (EYA4) was found to recapitulate the phenotypes of impaired cardiac contraction and heart failure seen in human patients [76]. A novel Z-disk protein nexilin (encoded by NEXN) was identified in zebrafish and has been linked to DCM in humans and zebrafish [77]. Zebrafish are suitable for modeling candidate mutations and variants. Several zebrafish DCM genes have a human ortholog, and a human TNNT2 mutation that causes hypertrophic cardiomyopathy (HCM) was modeled in an embryonic zebrafish [67]. Thus, the zebrafish model provides an opportunity to explore the genetic interactions and molecular mechanisms underlying DCM. Further, zebrafish cardiomyopathy models have been utilized to explore the therapeutic potential of Wnt/β-catenin signaling in cardiac hyperplasia. An anemia-induced zebrafish model of cardiomyopathy (tr265) was used and crossed with heat-shock-inducible transgenic fish to modulate the Wnt/β-catenin pathway. Increased activity of the Wnt/β-catenin pathway improved fish survival, probably providing a cardio-protective function via increasing cardiomyocyte hyperplasia [67]. Mutations in zebrafish cardiac troponin C (cTnC) were induced using the conditional expression of antisense RNA and phenotypes wherein cardiac chambers increased in size, and a lower ventricular ejection fraction was observed when compared to the control embryos [78]. Xr1 is a fragile X-related (FXR) gene family member that is expressed in striated and cardiac muscle. The knockdown of fxr1 using antisense morpholino revealed defect reduction in cardiac function as measured via high-speed video microscopy and showed a looping defect of the atrium [79]. A study by Dhandapani PS et al. discovered functional rare RAF1 mutations in the cohorts of South Indian, North Indian, and Japanese populations with a 9% prevalence of childhood-onset DCM cases. This group modeled these mutations in zebrafish and found that due to the altered kinase activity of the RAF1 mutation, the fish embryos showed a heart failure phenotype induced by AKT hyperactivation and rescued by rapamycin [80]. 

Zebrafish have also been used as models for studying a disease associated with changes in the cardiac thick filaments [81]. Cardiac thick filaments were isolated from zebrafish cardiac muscle, and the structural components were analyzed and reconstructed using electron microscopy. The rebuilt structure showed similarities to mammalian cardiac filaments, thus providing a model for understanding the roles of proteins such as cardiac myosin binding protein-C or titin that form these filaments. The Pkd2 mutant zebrafish showed low cardiac output and atrioventricular block, and mutant hearts displayed disturbed intracellular calcium cycling, contributing to heart failure [82]. Transcriptome analysis of zebrafish hearts identified the corresponding ortholog for the most-known genes for human DCM [70]. The application of forward genetics to zebrafish has identified many mutated genes that impact cardiac contractile function [21,22,83]. 

### 4.3. Arteriogenesis

Arteriogenesis has been studied in several models, including mice, but these models are limited by technical difficulties, as visualizing collateral vessel development in vivo is laborious [83]. The availability of mutants with arterial defects or permanently occluded aortas would provide better insight into developing a vessel. As models for understanding arteriogenesis, zebrafish fill this gap as they share the mechanisms of artery development found in mammals [84,85,86]. The role of ischemia in arteriogenesis is disputed, and mouse models have failed to resolve this question. A zebrafish arteriogenesis model was developed because a zebrafish embryo gains sufficient oxygenation via diffusion to prevent ischemia in response to arterial occlusion [84]. A laser induced aortic occlusion, in zebrafish showed collateral vessel development in the absence of ischemia.

Zebrafish have been used to study the role of N-ras signaling during endothelial differentiation in vertebrates. The zebrafish lmo2 promoter was used to express a constitutively active version of human N-Ras, specifically in endothelial and hematopoietic cells. Activated N-Ras signaling disrupted the balance of arterial–venous specification. This model effectively provides new insights into the pathogenesis of congenital human vascular disease and tumorigenic angiogenesis [87,88]. The availability of zebrafish mutants with arterial defects or permanently occluded aorta would help us understand arteriogenesis, as they share the mechanisms of artery development found in mammals [88]. One such mutant is the zebrafish gridlock mutant. This mutant displayed an incompletely formed lateral aorta and is deficient in circulation. ERK signaling is important to arteriogenesis in zebrafish. Activation of ERK1/2 in gridlock mutants by partially inhibiting PI3K activity restores the distal arterial circulation [89].

Further, the role of ischemia in arteriogenesis is disputed, and mouse models have failed to resolve this question. A zebrafish arteriogenesis model is feasible because the embryo gains sufficient oxygenation via diffusion to prevent ischemia in response to arterial occlusion [84]. It was found that collateral vessels could develop in the absence of ischemia. This study underscores zebrafish’s utility as a collateral vessel development model, allowing a novel approach to the study of arteriogenesis.

### 4.4. Thrombosis

Platelets play a crucial role in primary homeostasis and facilitate thrombus formation; thus, they are a primary pharmaceutical target, as decreasing thrombus formation can minimize the incidence of myocardial infarction and stroke. Understanding the mechanisms involved in platelet function and thrombus formation is imperative to identify drug targets for vascular disease management. Though mouse knockout models have provided insights into characterizing individual genes, their use is limited by requirements for substantial investments and manpower and the possibility of being unable to guarantee a relevant phenotype. In this scenario, the zebrafish (*Danio rerio*) is being explored as a model for studying thrombosis [90,91,92,93,94,95]. The zebrafish thrombocyte is the functional equivalent of the mammalian platelet. The ability to induce localized thrombosis by targeted laser injury [92], along with the transparency of the zebrafish embryo, enables real-time observation of arterial or venous thrombus formation after an endothelial injury. Forward genetic studies are underway to identify genes determining the thrombotic response to such arterial injuries [92]. Liu Yang et al. generated a zebrafish model of anti-thrombin III (AT3) deficiency by adopting a reverse genetics approach using zinc-finger nucleases [93]. Homozygous at3 zebrafish mutants showed intra-cardiac thrombosis between 2 and 7 months of age. This study uncovered the mechanism behind the at3 mutant phenotypes and suggested a conserved role of the At3 function in zebrafish.

In vivo reverse genetics functional screening of new platelet genes in zebrafish is easy. A comparative transcript study of platelets and their precursor cells, megakaryocytes, nucleated blood cell elements, endothelial cells, and erythroblasts revealed several unique platelet membrane proteins with unknown roles in thrombus formation. Reverse genetics was used to study five genes in a laser-induced arterial thrombosis model. BMP, BAMBI, and LRRC32 may promote thrombus development. Many large-scale screening programs and genome-wide association and “omics” studies have found genes and loci involved in platelet production and physiology under normal and pathologic settings. Such a resource requires enhanced model systems for in vivo gene functional assessment and the validation of new anti-thrombotic advances [94,95,96,97,98,99]. Zebrafish models have the potential to facilitate these gene discovery advancements and thus can be utilized for functional validation and in therapy-based studies to address thrombus formation.

Understanding the mechanisms involved in platelet function and thrombus formation is imperative to identify drug targets for vascular disease management. Though mouse knockout models have provided insights into characterizing individual genes, their use is limited by requirements for substantial investments and manpower and the possibility of being unable to guarantee a relevant phenotype. In this scenario, the zebrafish (*Danio rerio*) is being explored as a model for studying thrombosis [99,100,101,102]. The zebrafish thrombocyte is the functional equivalent of the mammalian platelet. Thrombocyte proliferation and maturation during zebrafish embryogenesis can be easily tracked using the integrin CD41/CD61 subunit, a platelet-specific marker [100]. The ability to induce localized thrombosis via targeted laser injury [95], paired with the transparency of the zebrafish embryo, enables the real-time observation of arterial or venous thrombus formation after an endothelial injury. Forward genetic studies are underway to identify genes determining the thrombotic response to such arterial injuries [103].

The zebrafish is amenable to the functional screening of novel platelet genes. For example, the function of five of these genes was studied in a laser-induced arterial thrombosis model using a reverse genetics approach, as reviewed by Gut et al. 2017 [104]. Putative roles of BMP, activin membrane-bound inhibitor homolog (BAMBI), and Leucine-rich repeat-containing protein 32 (LRRC32) in promoting thrombus formation were identified. It was found that Discoidin, CUB, and LCCL domain-containing protein 2 (DCBLD2) and endothelial cell adhesion molecule (ESAM) serve to inhibit thrombus formation. Such information on molecules and genes that either support or inhibit platelet-led thrombus formation is essential for designing targets for therapeutic intervention for thrombosis management. In a separate study, Zhu et al. utilized zebrafish to assess anti-thrombotic drugs. This study tested six human antithrombotic drugs (aspirin, clopidogrel, diltiazem hydrochloride, xuanshuantong, salvianolate, and astragalus) in a zebrafish thrombosis model. A larval zebrafish thrombosis model was designed and validated for in vivo studies for quick antithrombotic medication screening and efficacy assessment [103]. Several large-scale screening programs and genome-wide association and “omics” studies have discovered genes and loci associated with the formation or physiology of platelets under normal and pathologic conditions. Such a resource necessitates the availability of improved model systems that permit in vivo functional assessment of these genes and the validation of recent innovations in anti-thrombotic [99,100]. The Zebrafish model has the potential to facilitate these gene discovery advancements and thus can be utilized for functional validation and therapy-based studies to address thrombus formation. Recently, Zhu et al. have shown that Wuliangye prevents and reduces thrombosis via changing metabolic and signaling pathways associated with platelet aggregation and adhesion, oxidative stress, and the inflammatory response [105].

### 4.5. Modeling Cardiac Rhythm Disorders in Zebrafish

Zebrafish have been widely employed in arrhythmia research [31,106,107,108,109,110] owing to the success of multiple genetic screens, resulting in mutants resembling arrhythmic conditions [21]. The classic genome-wide screening techniques that have been used make genetic models of organisms such as yeast, nematode, or drosophila, powerful tools in the dissection of cell signaling and other phenotypes, have not proven feasible for many physiologic traits, including cardiac electrophysiology [111,112,113,114]. Zebrafish models have facilitated the rigorous exploration of arrhythmia mechanisms and allowed systematic approaches to discovering and testing novel drugs or other interventions [113,114,115,116,117]. The earliest electrophysiological phenomena in cardiac development are accessible in zebrafish. Calcium and voltage transients can be easily recorded in the cardiac primordia before midline migration and fusion into the primitive heart tube [116]. The physiological events in zebrafish are tied closely with cardiac development and can be visualized in vivo (Table 3) [113]. Zebrafish embryonic and adult heart rates and AP and ECG morphology closely resemble those of humans [112,113,114,115]. Thus, the zebrafish is a relevant research model for investigating ion channel disorders associated with abnormal repolarization or Ca^2+^-modulated arrhythmias [31]. Humans frequently experience atrial fibrillation (AF). Sequencing of AF patients revealed a mutation in the BMP antagonist gremlin-2 (GREM2). Zebrafish functional modeling showed that GREM2 regulates BMP signaling to influence cardiac laterality and atrial development. Live heart imaging of zebrafish embryos overexpressing wild-type or mutant GREM2 showed decreased atrial cardiomyocyte contraction rates. Zebrafish can be utilized to study the etiology of human AF and identify novel regulators [113,118]. Many zebrafish lines with ion channel mutations are being exploited as vertebrate models for long- or short-QT syndrome [35]. For this, a transgenic zebrafish arrhythmia model with the human SCN5A-D1275N mutation was developed. Mutations in the cardiac sodium channel SCN5A-D1275N in humans and mice cause atrial and ventricular arrhythmias. SCN5A-D1275N transgenic zebrafish exhibited bradycardia, conduction-system defects, and early death. ECG measures for embryonic and larval zebrafish have been refined to assess QT-prolonging medications, including terfenadine, verapamil, and haloperidol. ECG recording may be used to investigate drugs for cardiac cycle abnormalities and downstream effects, including heart block and arrhythmias in zebrafish larvae and adult [33,34]. The lack of high-throughput functional assays hinders long-QT syndrome genetic testing for determining gene variant pathogenicity correctly. Zebrafish assays have been established to investigate the capacity of known long-QT syndrome hERG1 (KCNH2) mutations and polymorphisms to achieve proper repolarization in kcnh2-knockdown embryonic zebrafish. Genetic modeling and the molecular dissection of human rhythm disorders, as well as the study of electrophysiological features and screens to identify key molecules and pharmacological targets, can be easily achieved in zebrafish models.

**Figure 2:**
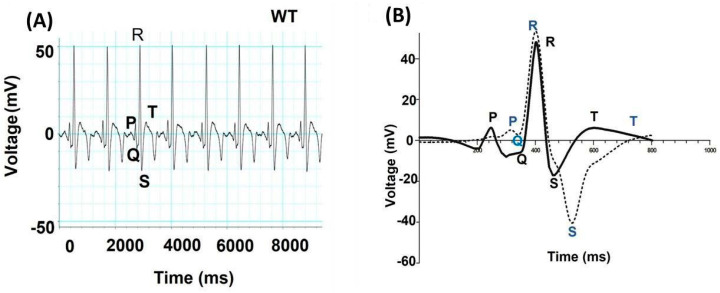
Adult zebrafish electrocardiogram (**A**) Electrocardiogram recordings of wild-type (*bh^+/+^*) zebrafish. X-axis represents time in milliseconds (ms), and Y-axis represents voltage in millivolts (mV). (**B**) Graph showing an overlay of wild type and *bh^-/-^* mutant ECG recordings (Regular line and dashed line represents the ECG profiles of wild type and *bh^-/-^* fish, respectively). P, Q, R, S & T represent regular ECG waves. The image in the figure were adapted from the author’s publication [119].

The human and two-chamber zebrafish hearts have similar baseline electrocardiograms despite their anatomical differences. Their P, QRS, and T waves; QT interval duration; and sluggish heart rate were like those of humans (Figure 2). Like human QT duration, zebrafish QT duration is heavily dependent on cardiac frequency and must be corrected for heart rate (QTc) [35]. Many pathological ECG abnormalities in human hearts can be replicated in zebrafish. The sequencing of atrial fibrillation (AF) patients revealed a variance in the BMP antagonist gremlin 2. Functional modeling of GREM2 in zebrafish revealed that it affects cardiac laterality and atrial differentiation by regulating BMP signaling. Using live heart imaging of zebrafish embryos that over-expressed wild-type or variant GREM2, slower cardiac contraction rates were recorded, specifically in atrial cardiomyocytes. The implications of BMP signaling in human AF were uncovered, demonstrating that zebrafish can be used to investigate mechanistic insights into the pathogenesis of this disease and that novel regulators can be identified [118]. Several zebrafish lines with ion channel mutations are now being used as vertebrate models for understanding long- or short-QT syndrome [105]. 

Towards this end, a transgenic zebrafish arrhythmia model with the human SCN5A-D1275N (cardiac sodium channel) mutation was created. The mutations in SCN5A-D1275N are associated with various cardiac phenotypes, including atrial and ventricular arrhythmias in humans and mice. Transgenic zebrafish expressing the SCN5A-D1275N mutation exhibit bradycardia, conduction-system abnormalities, and premature death. These phenotypes could be rescued using both human and zebrafish mRNA, suggesting a conserved role of SCN5A in vertebrate heart rhythms. Thus, zebrafish in vivo models for human genetic variants offer a functional assessment of cardiac ion channel genes and the mechanisms involved [35].

ECG measurements have been optimized for embryonic and larval zebrafish to quantify the effect of QT-prolonging drugs such as terfenadine, verapamil, and haloperidol. This circumvents the usage of mammals in ECG-based preliminary screening to detect cardiac dysfunction [34]. A significant problem with genetic testing for long-QT syndrome is the lack of robust functional assays with which to accurately decipher the pathogenicity of identified gene variants in a high-throughput manner. To address this problem, a zebrafish assay was developed to test the ability of known long-QT syndrome hERG1 (KCNH2) mutations and polymorphisms to attain normal repolarization in a kcnh2-knockdown embryonic zebrafish. Kcnh2 is the only repolarizing ion channel in the embryonic zebrafish ventricle. This assay could correctly distinguish a benign variant from a disease-causing variant. The cited study demonstrated the utility of zebrafish in designing cardiac assays that can serve as additional tools for testing the pathogenicity of gene variants in cardiac disorders. Another group developed a larval zebrafish model to quantify chronotropic, inotropic, and arrhythmic effects together with blood flow and vessel diameter. Cardio stimulants (adrenaline and theophylline) and negative chrono/inotropes (cisapride, haloperidol, terfenadine, and verapamil) were evaluated in zebrafish, and cardiac chamber beat frequencies were recorded. Such a model thus has implications for higher-throughput drug safety and efficacy applications [120]. Therefore, genetic modeling and molecular dissection of human rhythm disorders, the study of electrophysiological features, and screens to identify key molecules and pharmacological targets can be easily achieved in the zebrafish model. APs from adult zebrafish ventricular myocytes were recorded for the first time by Brette et al. [117] and further characterized by Nemtsas et al. (2010) [115]. These pioneering works showed that the resting membrane potential and AP amplitude were similar in ventricular myocytes isolated from humans and zebrafish. Like humans, the zebrafish AP had a plateau rather than a spike or phase 1. Zebrafish have a shorter action potential duration (APD) than humans due to their higher resting heart rate. Like humans and other bigger mammals, APD shortens as stimulus frequency increases due to a shorter plateau phase. Thus, zebrafish ventricular APs mirror those of big mammals, including humans, unlike mice and rats’ triangular and nearly rate-independent APs.

### 4.6. Limitations of Zebrafish as a Cardiovascular Disease Model

#### 4.6.1. Simplified Cardiovascular Anatomy

The zebrafish cardiovascular system is simpler than that of mammals, lacking certain structures such as a coronary circulation system. While it allows for studying basic cardiovascular processes, some aspects of human cardiovascular diseases may not be fully recapitulated in zebrafish models.

#### 4.6.2. Limited Behavioral Studies

The behavioral aspects of cardiovascular diseases, including exercise tolerance and complex cardiovascular responses to stress, are challenging to study in zebrafish. This limitation underscores the need for complementary studies using mammalian models to capture the full spectrum of cardiovascular disease manifestations.

#### 4.6.3. Temperature Sensitivity

Zebrafish are ectothermic, and their physiological processes are influenced by temperature. This temperature sensitivity can pose challenges when attempting to model certain aspects of cardiovascular diseases that are temperature-dependent.

## 5. Summary

Since their introduction, Zebrafish have been used extensively to investigate the circulatory system. In recent decades, zebrafish have become an essential model for heart development, illness, and regeneration. The zebrafish is a great model for disease causes and potential treatments due to its transgenic availability and amenability to genetic manipulation and in vivo chemical and genetic screening. With its unique ability to regenerate, the zebrafish heart offers a great chance to learn how to battle fibrosis and cardiomyocyte loss with a natural origin. Finding the molecular mechanisms governing these activities will lead to HF and other heart disease treatments. Recent instances show that this animal model can be used to find new cardiomyopathy susceptibility genes and intriguing treatment targets despite technical obstacles. In the coming years, gene-editing tools will improve, allowing those involved in precision medicine to understand this disease’s biology and find cures.

## 6. Metabolic Disease Models

Zebrafish represent an intriguing animal model for comprehending the pathophysiology of metabolic disorders in humans and pinpointing prospective treatment avenues [121] (Figure 1). The metabolic features of zebrafish are comparable to those of humans, so they can be used to supplement information derived from other model organisms, such as rats. This possibility has been amply demonstrated by recent research [122,123,124,125] that found that medications licensed for treating human metabolic disorders also worked well in a zebrafish model. Numerous potential human disease genes and loci linked to metabolic disease have been found thanks to genome-wide association studies (GWAS) and, more recently, whole-exome and whole-genome sequencing [126]. How these genes work and how their malfunction impacts pathophysiology are the current problems in this regard. Zebrafish have been used to effectively examine variant function in vivo at a reasonably high throughput in various disease areas. In recent work, platelet production, which has implications for hematological disease, was discussed [127]. These advantages of zebrafish can be used to make comparable advancements in the field of metabolism. In this section of our review, we focus on the use of zebrafish as an animal model for metabolic disease and their pros and cons in this application. We also examine the latest developments in modeling the associated disorders of metabolic syndrome—obesity, diabetes, fatty liver disease, and atherosclerosis—using zebrafish.

Recently, zebrafish have become popular models for metabolic disorders [128]. This review aims to provide an overview of the strengths and limitations of the zebrafish metabolic disease model, highlighting its contributions to our understanding of metabolic disorders. Though more research is required, evidence supports the idea that zebrafish have similar control mechanisms due to similarities in the peripheral nervous system’s development and the way drugs that affect the serotonergic or adrenergic systems can modify glucose metabolism [129,130]. Zebrafish metabolism research has advanced slowly compared to the cardiovascular discipline due to these and other factors. Table 4 presents some of the established metabolic disorder models: acute hyperglycemia [131,132,133,134], chronic hyperglycemia [133,134,135,136,137,138], genetically induced [139,140,141], and obesity [142,143,144]. We discuss select zebrafish-based metabolic disease models below.

### 6.1. Obesity

Over time, accumulating significant quantities of dietary triglycerides in adipose tissue leads to obesity. While obesity is increasingly being recognized as a disease in and of itself, a person’s genetic history, gender, and lifestyle and environmental factors all have a significant impact on their likelihood of developing diabetes. For instance, certain people who are extremely fat but deemed healthy in terms of blood lipid profiles and insulin resistance, glucose regulation, and cardiovascular parameters are known as “healthy obese” people. It is believed that a significant factor in preventing difficulties linked to obesity is a strong ability to store neutral lipids and prevent systemic inflammation instead of exposing peripheral tissues to triglycerides, cholesterol, and fatty acids [145,146,147]. Zebrafish are used to study genetic and dietary factors that control the growth and plasticity of adipose tissues, as well as mechanisms of lipid absorption, packing, transport, and inflammatory responses to aggressive lipid moieties [148,149]. The underlying principles of adipose formation in zebrafish are like those in humans. Adipocytes are first seen in larvae soaked in the neutral lipid dye nile red approximately 7 days after they start feeding, whereas no adipocytes form in animals that are denied nourishment [150]. During the first four days of development, the yolk sac stores and synthesizes lipids [151], but its anatomical shape and energy substrate packing are not much like those of human adipose tissue. At around 30 dpf, when the energy is no longer entirely used for somatic growth, subcutaneous and visceral fat pads anatomically resembling human adipose tissue first develop [150,152,153].

Studies on genetic gain and loss of function have shown that numerous genetic mechanisms influencing adiposity in zebrafish are like those in mammals [154,155]. Nevertheless, deleting leptin signaling, one of the most researched signaling pathways that controls body fat mass, does not demonstrate a conserved function. When compared to healthy individuals, human patients with mutations impacting leptin signaling exhibit excessive eating tendencies, are morbidly obese, and have lower fertility. Similar behaviors are observed in mouse models; after eating, insulin stimulates the release of leptin, which is strongly expressed in mammalian adipocytes [156]. Zebrafish lacking the leptin receptor lepr, on the other hand, exhibit normal growth, fat accumulation, eating patterns, and fertility [157]. Zebrafish fat virtually never expresses the lep gene, but their liver experiences a rise in mRNA levels when they fast.

Zebrafish research has yielded genuinely innovative discoveries in the field of obesity [158,159]. Recent research, for instance, has uncovered a mechanism of PLXND1, a gene linked in population genetics studies to variations in the distribution of fat between the hips and waists in humans and an elevated risk for type 2 diabetes mellitus (T2DM) [160]. While Plexin D1 deletion in mice results in embryonic death before the development of visceral fat [161], Plexin D1/zebrafish is accessible for investigation. Interestingly, visceral fat depots in the zebrafish mutant are hyperplastic, with a reduced capacity to store lipids [161]. As visceral fat tissue could not grow under high-fat diet (HFD) conditions, surplus lipids had to be redistributed to subcutaneous depots. It was demonstrated that modifying the extracellular matrix surrounding the cell is the fundamental mechanism behind this process. This work offers an intriguing set of in vivo and ex vivo adipose tissue-imaging techniques that could be of great value for similar investigations in the future, in addition to revealing novel activities of plxnd1 [161,162].

Though adipose tissue is absent in the early stages of embryonic and larval zebrafish development, other aspects of lipid metabolism can be studied; for instance, the zebrafish gastrointestinal system is anatomically and functionally similar to the mammalian system [163,164]. One way to see lipid uptake and processing in enterocytes is to feed fluorescently tagged lipid moieties to zebrafish larvae. Using this technique, forward genetic search revealed a fat-free mutant, whose reduced processing of cholesterol and phospholipids was accompanied by unchanged intestinal morphology [164]. Later research revealed that this fat-free mutant is responsible for expressing Ang2, a conserved member of the Golgi-associated retrograde protein (GARP) complex, which controls autophagy, endosomal cholesterol trafficking, and lysosomal enzyme sorting [165,166].

### 6.2. Non-Alcoholic Fatty Liver Disease

Regardless of alcohol intake, nonalcoholic fatty liver disease (NAFLD) is the most common lipid storage disease of the liver and is mainly caused by high-fat diets and foods high in carbohydrates [167]. It is regarded as a consequence of obesity and affects more than 30% of the general population, as well as an even higher percentage of obese people. It is treatable via diet, but if left unchecked, it puts the body at risk for developing cirrhosis, hepatocellular carcinoma, and non-alcoholic steatohepatitis (NASH) [168]. It is unclear which hereditary variables cause benign nonalcoholic fatty liver disease (NALFD) to develop into inflammation, fibrosis, and, ultimately, malignant liver cancer [169]. The benefit of studying NAFLD in zebrafish is that whole-mount Oil Red O staining for neutral lipids allows for the visualization of steatosis in larvae [170,171]. Zebrafish can be fed lipid-rich meals, such as an egg yolk emulsion [172] or Artemia brine shrimp, after feeding begins at 4 dpf. As an alternative, steatosis has been induced via fructose-based or ketogenic diets [173]. Because the larval liver is large enough to be dissected easily, samples can be used for transcriptome, histological, and biochemical investigations [172]. Further, several transgenic lines are available for observing hepatic cell types, such as hepatocytes, biliary cells, endothelial cells, and stellate cells [174,175]. Adult zebrafish can be fed certain diets for an extended period, and the lipid content of their livers can be assessed using metabolomics or traditional enzymatic techniques [173,176]. 

Large-scale forward genetics screens once more yielded the first zebrafish models of NAFLD, indicating that significant insights into disease mechanisms can be attained by methodically searching for mutations that cause NAFLD and examining their involvement in this illness’s course [176,177,178]. An ENU mutagenesis screen revealed 19 novel hepatomegaly-characterized mutant lines, most of which showed neutral solid lipid staining. Numerous mutants have distinct histological pathological characteristics, varying from mild cases of micro- or macrovesicular steatosis to severe indications of nonalcoholic steatosis, which include hepatocyte necrosis and ballooning [179]. Cloning the genes impacted by these mutations will probably provide crucial information on how chronic liver disease develops.

### 6.3. Atherosclerosis

Zebrafish possess features that aid in the study of atherosclerosis, such as the ability to detect lipid deposits inside blood vessels in vivo. According to a series of landmark investigations by Miller’s team, wild-type zebrafish continuously fed high-cholesterol diets are more likely to develop histological alterations resembling human atherosclerosis than rodents [164,180]. Therefore, research on murine atherosclerosis relies on a sensitive background, like ApoE/E animals [181]. The fact that the ortholog of the human cholesteryl ester transfer protein (CETP) remains preserved in zebrafish but has vanished from rat genomes provides an evolutionary explanation for this anomaly. Hence, zebrafish experience atherogenic events more frequently than humans because cholesteryl esters in fish are redirected from protective HDL cholesterol particles to “bad” LDL. Miller’s group also demonstrated that single-chain monoclonal antibodies coupled with GFP can be used to visualize the oxidation of LDL by malondialdehyde, thereby exploiting zebrafish’s advantages for in vivo imaging [180]. Moreover, tracking and measuring the recruitment of macrophages to vascular lesions can be used to develop novel treatment approaches.

Interestingly, the human liver X receptor (encoded by LXRA and LXRB) has functionally conserved activities in zebrafish [182]. In contrast to mice, zebrafish have a single lxr ortholog (encoded by lxra/nr1h3), making it possible to determine its physiological roles through comparatively simple gain- and loss-of-function tests. Zebrafish with the lxra// knockout allele remain alive. However, they exhibit high blood and liver cholesterol levels following prolonged adherence to a high-fat diet (HFD), like the cholesterol intolerance shown in Lxra// and Lxrb// double knockout mice. The authors also showed through overexpression experiments that increased Lxr function, particularly in enterocytes, could have the opposite impact, i.e., a significant slowdown in the accumulation of cholesterol in the blood and liver in response to HFD [183]. From a mechanical perspective, it was discovered that Lxr triggers an acyl-CoA synthetase (encoded by acsl3a) that breaks down ingested lipids and directs them toward lipid droplets for storage rather than rapid release into the bloodstream. This finding has significant clinical implications because multiple studies suggest that postprandial elevations in cholesterol are the primary cause of atherosclerosis. Statins, which prevent the liver’s de novo manufacture of cholesterol, do not, however, mainly successfully regulate the postprandial dynamics of cholesterol.

### 6.4. Diabetes

The Zebrafish has been explored as a suitable model for diabetic research [184,185]. Zhang et al. demonstrated that a new diet-induced obesity (DIO) zebrafish has been shown to display higher blood glucose levels after fasting than normally fed ones. This hyperglycemic zebrafish is a helpful model for T2DM, measuring glucose tolerance, insulin production, and glycaemia responsiveness to human anti-diabetic medications. In addition, liver–pancreas RNA-seq research shows that T2DM zebrafish share human pathogenic pathways [185]. When responding to damage and metabolic stresses, a zebrafish’s beta cell mass is remarkably malleable [135,186]. Zebrafish research has primarily focused on the dissection of the genetic cues that govern the formation, maturation, and flexibility of functioning beta cells. This work is beginning to yield significant insights for therapies for replacing and regenerating beta cells. In this context, several different strategies are being investigated, ranging from the in vitro differentiation of mature beta cells from induced pluripotent stem cells to the in vivo restoration of sufficient numbers of functional beta cells through progenitor pools, non-beta cells, or remaining beta cells [186,187,188]. Because each progenitor and cell type within the islet can be seen and tracked over time in vivo using fluorescent reporter proteins until the formation of a mature pancreas throughout the embryonic, larval, juvenile, and adult stages, zebrafish are anatomically well suited for this purpose [189,190,191,192,193,194,195,196,197,198,199,200,201]. Zebrafish have a single primary islet located in the pancreas’s head in addition to numerous minor islets spread throughout this organ [191,192]. Once again, forward genetic screens provided the first research indicating that zebrafish may be used to analyze the genetic network controlling endocrine pancreas development [194]. A line with a mutation in the homeobox gene vhnf1, which prevents normal expression of pdx1, a transcription factor gene required for the induction of the insulin gene, was found in an insertional mutagenesis screen [140]. Lesions in these genes are known to induce a subtype of monogenic diabetes called mature-onset diabetes of the young (MODY) type V for VHNF1 and type IV for PDX1.

Hence, the significance of these findings with respect to this condition became evident. It was subsequently demonstrated that pdx1 deletion in zebrafish results in a drop in insulin levels, a disruption of glucose homeostasis, and a reduction in beta cell numbers [141]. These days, drug screens are performed to identify small compounds that stimulate or obstruct distinct stages of endocrine lineage maturation and specification. Ultimately, these chemicals may be employed to boost the yield of mature beta cells from iPS cells in vitro or stimulate precursor populations that can produce new beta cells in vivo [194]. Using the double transgenic lines Tg(Tp1:hmgb1-mCherry) and Tg(pax6b:GFP), a screen demonstrated that this method was able to identify pax6b-positive progenitor cells inside the intrapancreatic duct [194]. The intrapancreatic duct cells were marked by a Notch activity reporter carried by Tg(Tp1:hmgb1-mCherry) animals. When Notch signaling is suppressed, these progenitor cells are activated, becoming beta cells.

### 6.5. Limitations of Zebrafish as a Metabolic Disease Model

Researching zebrafish energy metabolism uncovers additional technological difficulties. For instance, zebrafish find it challenging to perform standard tests for humans, such as hormone and glucose tolerance and insulin test. Because the macro- and micronutrient requirements of humans and zebrafish differ significantly, it is challenging to convert dietary data from interventions for zebrafish into human diet guidelines. Monitoring a zebrafish’s food intake in a tank is challenging, yet food intake is strongly correlated with growth rate, affecting adipocyte production. This is because zebrafish, throughout young adulthood, prefer somatic growth over body fat deposition. When raising a family of wild-type siblings, this issue becomes apparent since there can be significant variations in growth rates and adiposity due to competition for food, genetic background variances, and other variables. In this poikilothermic mammal, temperature also influences development, metabolic rate, and body fat composition.

Additionally, the microbiome may have an impact on metabolism, which is not taken into account in zebrafish facilities. In contrast to traditional or even specialist germ-free (SPF) mouse housing, water circulation carries the risk of many more possible pathogens. Zebrafish have been shown to have a core microbiome. Currently, several groups are investigating how alterations in the microbiota of the zebrafish gut, either those naturally occurring or caused by infections, affect lipid and glucose homeostasis, an idea that is gaining much attention with respect to mammals. The regulation of systemic metabolism in zebrafish through the innervation of glands such as the thyroid, chromaffin cells, and pancreas, or that of metabolic organs such as enterocytes, the liver, and adipose tissue by sympathetic and parasympathetic inputs, is another aspect that is still mainly unknown.

### 6.6. Mammalian-Specific Aspects

While zebrafish share many genetic similarities with humans, they lack certain mammalian-specific aspects of metabolism. Therefore, findings from zebrafish studies need to be validated in mammalian models and, ultimately, in human clinical trials to ensure the relevance of the results.

### 6.7. Limited Tissue Complexity

Zebrafish organs are less complex than those of their mammalian counterparts. This limitation may restrict zebrafish models’ ability to fully capture the intricacies of certain metabolic diseases, especially those involving highly specialized tissues or organs.

### 6.8. Limited Behavioral Studies

Behavioral aspects of metabolic diseases, such as diet-induced behaviors and complex feeding patterns, are challenging to study in zebrafish. This limitation may impede a comprehensive understanding of the behavioral aspects associated with metabolic disorders.

## 7. Summary

Many metabolic diseases have been modeled in zebrafish. Despite its limitations, the zebrafish model has been successfully used to visualize dynamic cell processes and conduct high-throughput small-molecule screens for illnesses impacting β-cell function. The zebrafish models for obesity, NAFLD, and atherosclerosis are poised to exploit this model system’s benefits and replicate diabetes developments. The zebrafish model could attain its full potential in metabolism research via the creation of fluorescence reporter lines for adipose tissue and hypothalamus cell types and enhanced insulin-signaling techniques. Zebrafish-based metabolic disease models provide several fascinating possibilities. We hope the zebrafish system will help us understand metabolic disease development and progression and find new therapy options.

## 8. Conclusions

In the last two decades, the zebrafish model has emerged as a powerful model system for studying cardiac development, cardiac functions, and human heart disorders. Several zebrafish genetic screens have yielded a collection of cardiac mutants affecting several processes during early heart development and chamber morphogenesis. It is easier to follow the details of cardiac development by combining this cardiac mutant with high-resolution and advanced (3D and 4D) imaging tools. Moving forward from development to function, there has been a paramount development in the genome-editing tools in zebrafish biology. Specifically, targeted gene mutations can be easily created in zebrafish germlines by applying zinc-finger nucleases (ZFNs) and recently developed TALENS and CRISPR technologies. These tools have overcome the limitation of being unable to perform site-directed mutagenesis in zebrafish. The progress in gene-editing and targeting technologies has provided a scope for the functional studies of genes and variations implicated in CVD in zebrafish. This fish model is being utilized for investigating adult cardiac disorders as it provides the advantage of enabling the study of adult heterozygous fish to obtain insights into the progression of cardiac disease and its pathogenesis.

Further, zebrafish have provided significant contributions in the field of drug discovery, and it is expected that these efforts will soon generate a library of compounds with cardiovascular disease-monitoring properties. This is strongly supported by pharmacological screens that have yielded compounds beneficial for managing or treating cardiovascular disorders. Altogether, zebrafish cardiovascular research has provided evidence filling the knowledge gap in understanding human and other mammalian cardiac development and disorders. The current review provides an overview of the existing tools and resources available in zebrafish research and discusses the various advancements in this field.

The zebrafish cardiovascular disease model has proven to be an asset in cardiovascular research, offering unique advantages in terms of genetic conservation, transparency, and ease of genetic manipulation. While this model has its limitations, particularly in reproducing the complexity of the mammalian cardiovascular system, its contributions to understanding basic cardiovascular processes and facilitating drug discovery are undeniable. As technology continues to evolve, the zebrafish model will continue to be a powerful tool for exploring the causes of cardiovascular diseases and developing innovative therapeutic strategies. On the other hand, the zebrafish metabolic disease model offers a powerful platform for investigating the genetic and molecular mechanisms underlying metabolic disorders. Its genetic similarity, transparency, reproductive rate, and cost-effectiveness advantages make it a valuable tool for high-throughput screening and large-scale studies. However, researchers should be mindful of this model’s limitations, emphasizing the need for complementary studies in mammalian models and clinical settings to validate and translate findings into human therapeutic applications. As technology advances and researchers refine their approaches, the zebrafish model will likely continue playing a fundamental role in evolving our understanding of metabolic diseases. 

## 9. Future Directions

The future of zebrafish research in cardiovascular and metabolic studies offers exciting possibilities, driven by technological advancements, interdisciplinary approaches, and the ongoing expansion of our understanding of these complex biological processes. Below, we provide some potential future directions for zebrafish research in cardiovascular and metabolic research.

### 9.1. Precision Medicine and Personalized Therapies

Leverage zebrafish models for understanding the genetic basis of individual variation in cardiovascular and metabolic responses. Explore personalized therapeutic interventions based on genetic and molecular profiles.

### 9.2. Functional Genomics and Systems Biology

Combine high-throughput genetic screens with advanced imaging technologies to uncover novel regulators of cardiovascular and metabolic pathways. Apply systems biology approaches to integrate multi-omics data to understand biological networks comprehensively.

### 9.3. Single-Cell Analysis

Adopt single-cell RNA sequencing and other single-cell technologies to dissect cellular heterogeneity within the heart and metabolic tissues. Investigate how individual cell types contribute to disease progression and response to therapies.

### 9.4. Live Imaging and 3D Models

Enhance real-time imaging techniques to capture dynamic processes in cardiovascular and metabolic tissues at cellular and subcellular resolutions. Develop advanced 3D culture systems that better mimic in vivo conditions for studying tissue-specific responses.

### 9.5. Integrative Models for Disease Complexities

Create multifactorial zebrafish models that better incorporate genetic, environmental, and lifestyle factors to represent the complexities of cardiovascular and metabolic diseases. Study comorbidities and interactions between different organ systems in the context of metabolic disorders.

### 9.6. Non-Coding RNA Biology

Explore the role of non-coding RNAs in regulating cardiovascular and metabolic processes. Explore the therapeutic potential of manipulating non-coding RNAs for disease intervention.

### 9.7. Environmental Influences and Nutritional Studies

Investigate the impact of environmental pollutants, diet, and lifestyle on cardiovascular and metabolic health using zebrafish models. Investigate the correlation between genetic susceptibility and environmental influences.

### 9.8. Drug Discovery and Screening

Continue to use zebrafish for high-throughput drug screening to identify novel therapeutic compounds for cardiovascular and metabolic diseases. Optimize preclinical drug testing by integrating zebrafish models into the drug discovery pipeline.

### 9.9. Functional Validation in Mammalian Models

Validate findings from zebrafish studies in mammalian models to bridge the translational gap and enhance the clinical relevance of discoveries.

### 9.10. Innovations in Genome Editing

Implement emerging genome-editing technologies to refine and enhance genetic manipulation’s precision in zebrafish models. Explore using base and prime editing for more targeted and controlled genetic modifications. These future directions underscore the potential of zebrafish research to contribute significantly to our understanding of cardiovascular and metabolic diseases, offering valuable insights and innovative solutions for improving human health.

### 9.11. Genomics in Cardiovascular Disease

Identification of Genetic Risk Factors: Genome-wide association studies (GWAS) leverage genomics to identify genetic variants associated with increased susceptibility to cardiovascular diseases. Precision Medicine: Genomic data enable the identification of individual genetic profiles, facilitating personalized treatment strategies based on genetic susceptibility and drug response.

### 9.12. Transcriptomics in Cardiovascular Disease

Gene Expression Profiling: Transcriptomics reveals gene expression patterns associated with various cardiovascular conditions, aiding in discovering prospective biomarkers and therapeutic targets. Alternative Splicing and Isoform Analysis: Examining alternative splicing events provides insights into the diversity of gene products and their relevance to cardiovascular health.

### 9.13. Proteomics in Cardiovascular Disease

Protein Profiling: Proteomics helps identify changes in protein expression, post-translational modifications, and protein–protein interactions associated with cardiovascular diseases. Biomarker Discovery: Identification of circulating protein biomarkers aids in the early diagnosis, risk stratification, and monitoring of cardiovascular diseases.

### 9.14. Metabolomics in Cardiovascular Disease

Metabolic Profiling: Metabolomics explores alterations in metabolite levels, providing insights into metabolic pathways associated with cardiovascular health and disease. Biomarker Identification: Metabolomic signatures are potential cardiovascular risk assessment and treatment-response-monitoring biomarkers.

### 9.15. Epigenomics in Cardiovascular Disease

DNA Methylation and Histone Modification Studies: Epigenomics involves investigating alterations in DNA methylation and histone modifications, shedding light on the epigenetic regulation of genes associated with cardiovascular diseases. Epi-transcriptomics: Studying RNA modifications enhances our understanding of the role of post-transcriptional regulation in cardiovascular health.

### 9.16. Integrated OMICS Approaches

Systems Biology: Integrating data from multiple OMICS platforms enables a holistic systems biology approach to unravelling complex connections linking genes, proteins, metabolites, and other molecular entities in cardiovascular diseases. Network Analysis: The construction of molecular interaction networks aids in identifying key nodes and pathways implicated in cardiovascular pathophysiology. Lastly, OMICS technologies, encompassing genomics, transcriptomics, proteomics, metabolomics, and other high-throughput approaches, are crucial in advancing our understanding of cardiovascular diseases (CVDs). These technologies provide comprehensive insights into the molecular mechanisms, genetic factors, and systemic changes associated with cardiovascular conditions.

Individualized Treatment: Pharmacogenomic studies explore genetic variations influencing drug metabolism and response, facilitating the development of personalized cardiovascular therapies. Adverse Drug Reaction Prediction: Genomic data help in predicting individual susceptibility to adverse drug reactions, enhancing drug safety in cardiovascular patients. Integrating OMICS technologies into cardiovascular disease research accelerates the discovery of molecular mechanisms, aids in identifying novel therapeutic targets, and contributes to developing personalized approaches for diagnosis and treatment in cardiovascular medicine.

## Figures and Tables

**Table 1:** Characteristic features of zebrafish.

Genetic Conservation and Transparency:	References
Zebrafish display a high degree of genetic similarity with humans regarding cardiovascular development and function. Their transparency during early development allows for real-time visualization of the developing heart and blood vessels, providing unparalleled insights into the molecular and cellular processes involved in cardiovascular diseases.	[13,14,15]
**High Reproductive Output:**	[16,17]
The prolific reproduction of zebrafish facilitates large-scale genetic and drug-related screening studies. Their ability to generate many embryos enables researchers to investigate various genetic and environmental factors influencing cardiovascular health, promoting the identification of novel therapeutic targets.	
**Conserved Cardiovascular Physiology:**	[21,26,27,28]
Zebrafish possess a cardiovascular system with remarkable similarities to mammals. Their two-chambered heart, like the human heart during early development, allows for investigating basic cardiovascular processes, including heart development, contractility, and blood vessel formation, in a simplified yet biologically relevant context.	
**Ease of Genetic Manipulation:**	[6,7,11,12]
The advent of advanced genetic tools, such as ZFN, TALEN, CRISPR/Cas9, has made targeted genetic modifications in zebrafish relatively straightforward. This ease of genetic manipulation enables researchers to model specific cardiovascular diseases, study the effects of gene mutations, and identify potential therapeutic targets.	
**Drug Discovery and Toxicity Screening:**	[32,33,34,35]
Zebrafish’s permeability to small molecules makes them amenable to drug delivery. This characteristic, coupled with the ease of monitoring their cardiac function, allows for efficient drug discovery and toxicity screening. Zebrafish models contribute to the preclinical evaluation of drug candidates for cardiovascular diseases.	

**Table 2:** List of selected zebrafish models of cardiac disease.

*Zebrafish Gene*	*Human Orthologue*	Model	Phenotype	Reference
*amhc*	*MYH6*	Mutant line	Hypoplastic atrium/enlarged ventricle/CM hyperplasia	[32]
*bag3*	*BAG3*	Mutant line	Ventricle hypertrophy, Trabecular density, Myofibril degeneration, Apoptosis	[36]
*scl4a1a*		Mutant line	Cardiomyopathy, myofibril degeneration, apoptosis, CM hypertrophy and hyperplasia	[37]
*cmlc1*	*MYL4*	Mutant line	Atrium enlargement, Disrupted sarcomeric structure	[38]
*vmhcl*	*MYH7*	Mutant line	Disrupted sarcomeric structure, Trabecular density, Enlarged ventricle	[39,40]
*gja3*	*GJA3*, *CX46*	Mutant line	Pericardial edema	[41]
*gtpbp3*	*GTBP3*	Mutant line	CM hypertrophy, Abnormal mitochondrial morphology, Disrupted sarcomeric structure	[42]
*vclb*	*VCL*	Gene trap line	Disorganized coronary development, Fibrosis, Epicardial/myocardial hyperplasia	[43]
*jupa*	*JUP*	Mutant lines	Cardiomegaly, Thin atrial/ventricular walls, Disrupted sarcomeric structure	[44]
*plcg1*	*PLCG1*		Normal myocardial ultrastructure	[45]
*jag2b*	*JAG2*	Ablation NC-derived CMs	Enlarged ventricle, CM hypertrophy	[46]
		Mutant line	Hypertrophic ventricle	
*tnnt2a*	*TNNT*	Mutant line	Abnormal sarcomeric assembly	[47]

**Table 3:** Zebrafish and Human Electrophysiology.

SL	Resemblances in Electrophysiology between Zebrafish and Humans	Reference
1	Similar long plateau phase	[112]
2	Presence of 0–4 phases	[112,113]
3	Ventricular AP has similar duration. The duration of zebrafish ventricular AP at 19 °C is like human ventricular AP at 37 °C	[114]
4	IK1 is present in atrial and ventricular myocytes	[112,114]
5	IK1 and IKr are the major repolarizing currents in both hearts	[115]
6	Similar fundamental current systems (INa, ICaL, and IK) are present in atrial and ventricular myocytes	[113,114]
7	Atrial and ventricular myocytes of the zebrafish heart have both T-type (ICaT) and L-type (ICaL) Ca^2+^ currents	[112,113,114]
8	The distinct QT intervals in ECG are similar in both	[112,114]
9	P, QRS, and T waves are clearly distinguishable in a zebrafish ECG	[112,114]

**Table 4:** Zebrafish metabolic disease models.

Disease	Method of Induction	References
Acute Hyperglycemia	Induced by intraperitoneal injection of D-glucose	[132,133]
Chronic Hyperglycemia		
1	Induced by the destruction of pancreatic cells	[134,135,136]
2	The induction of chronic hyperglycemia by dissolving D-glucose in fish water	[137]
3	Genetic induction	[138,139,140]
Obesity	Overfeeding models	[141,142,143]
